# SV40T/E6E7-Induced Proliferation Is Involved in the Activity of E2F3 in Bovine Mammary Epithelial Cells

**DOI:** 10.3390/ani12141790

**Published:** 2022-07-12

**Authors:** Yihui Zhang, Kang Zhan, Zixuan Hu, Guoqi Zhao

**Affiliations:** 1 Experimental Farm of Yangzhou University, Yangzhou University, Yangzhou 225009, China; yhz@yzu.edu.cn (Y.Z.); kzhan@yzu.edu.cn (K.Z.); huzixuan2021666@163.com (Z.H.); 2 College of Animal Science and Technology, Yangzhou University, Yangzhou 225009, China

**Keywords:** bovine mammary epithelial cells, E2F3, proliferation

## Abstract

**Simple Summary:**

A previous study demonstrated that SV40T does not require E2F1, E2F2, and E2F3 activators to induce proliferation in mouse embryo fibroblasts (MEFs). Our results showed that, at an early stage, primary bovine mammary epithelial cells (BMECs) lacking the E2F1 expression have the capacity to proliferate and show E2F2 and E2F3 slight protein levels. At a late stage, primary BMECs deficient for E2F3 completely abolish any proliferative ability and exhibit a severe cell senescence signal, although the E2F2 can be expressed at a late stage of primary BMECs. Compared with the late stage of primary BMECs, the BMECs immortalized by SV40T and E6E7 restored the protein level of E2F3. In conclusion, this study revealed a molecular mechanism where E2F3 controls the BMECs’ proliferation and senescence.

**Abstract:**

The E2F family of transcription factor is divided into activators and repressors that control cell proliferation. Bovine mammary epithelial cells (BMECs) can be immortalized using human papillomavirus 16 E6E7 (HPV16 E6E7) and simian vacuolating virus 40 large T antigen (SV40T). In addition, SV40T does not require E2F1, E2F2, and E2F3 activators to induce proliferation in mouse embryo fibroblasts (MEFs). However, we report that E2F3 activator is required to induce the proliferation of BMECs. Our results showed that, at an early stage, primary BMECs lacking the E2F1 expression have the capacity to proliferate and show E2F2 and E2F3 slight protein levels. At a late stage, primary BMECs deficient for E2F3 completely abolish any proliferative ability and exhibit a severe cell senescence signal, although the E2F2 can be expressed at a late stage of primary BMECs. Compared with the late stage of primary BMECs, the BMECs immortalized by SV40T and E6E7 restored the protein level of E2F3 and enhanced the CDK4, CDK6, cyclin D3, and CDK2 protein level, leading to proliferating robustly. Surprisingly, it was found that p53, p21^Cip1^, and p27^Kip1^ were upregulated in SV40T and E6E7-immortalized BMECs, relatively to primary BMECs. Notably, Cdc2 was almost expressed in primary BMECs. However, Cdc2 was elevated in BMECs immortalized by SV40T and E6E7. In conclusion, this study revealed a molecular mechanism where E2F3 controls the BMECs’ proliferation and senescence.

## 1. Introduction

Normal primary cells undergo only a limited number of cell proliferations in vitro before entering a non-dividing state known as replicative senescence [1]. This cell cycle progression and arrest are accompanied by E2F activation and increased levels of the p16^INK4A^, p53, and p21^Cip1^ proteins [2,3]. The E2F transcription factor family consists of nine structurally related members in higher eukaryotes. Three of them are activators: E2F1, E2F2, and E2F3a. Six others are divided among the inhibitors E2F3b and E2F4-8 [4]. Bovine E2F3 is not involved in the E2F3a and E2F3b from the NCBI database. However, *Drosophila melanogaster* has only a single E2F activator and repressor protein [5]. The E2Fs’ pathways are often disrupted in most human cancers, leading to increased E2F activity and an elevation of the E2Fs’ target genes [6]. In addition, SV40T and E6E7 are involved in E2Fs’ target pathways, suggesting an essential role in cell cycle progression and proliferation [7,8].

Previous studies have shown that E2Fs’ activators play a major role in cell survival and proliferation. Mutant mice derived from E2F1 and E2F2 double knockout are able to survive and develop to adulthood. Conversely, animals derived from E2F1, E2F3, and E2F2 double knockout died during early embryonic development, indicating the essential role of the E2F3 activator in mouse development [8,9]. Furthermore, mouse embryo fibroblasts (MEFs) that are deficient in E2F1 and E2F2 can still proliferate rapidly. Notably, the combined disruption of E2F1, E2F2, and E2F3 in MEFs severely impair the E2F target gene expression and cell proliferation [8,9]. However, in the absence of E2F1, E2F2, and E2F3 activators, epithelial stem cells, progenitors of the developing lens, and embryonic stem cells proliferate considerably and entry into the cell cycle occurs normally [10,11,12]. Remarkably, recent work suggests that SV40T can induce the transformation and proliferation of MEFs in the absence of E2F1, E2F2, and E2F3 activators [6]. These results indicate that alternative E2F activator pathways might regulate cell cycle entry and proliferation, and the process by which cells transform and proliferate is likely dependent on specific features of the cell type.

The Rb family controls cell proliferation and entry into the S-phase of the cell cycle by preferentially associating with three E2F activators. The E7 protein from the human papillomavirus, in addition to the SV40T antigen-induced transformation and proliferation, can disrupt Rb function. This, is turn, causes Rb to sequester E2F activators, thereby enhancing the expression of E2F target genes [13,14,15]. In addition, the SV40T antigen targets multiple cellular pathways to trigger cellular proliferation. SV40T disrupted Rb- and p53-dependent responses to enhance E2F1, E2F2, and E2F3, which drive quiescent cells to enter the S-phase [16]. We hypothesized that E2F activators may control the proliferation of BMECs. However, a recent study showed that the SV40T antigen induces proliferation and transformation in MEFs that lack the three E2F activators. In view of these previous findings, we investigated whether the proliferation of SV40T- and E6E7-induced bovine mammary epithelial cells (BMECs) requires E2F activators.

## 2. Materials and Methods

### 2.1. Cell Cultures

Primary and immortalized BMECs were obtained as previously described [17]. Pieces of bovine mammary tissue from healthy lactating Holstein cows (day 100 of lactation) were obtained from the experimental farm of Yang Zhou University. These mammary glands were used to obtain primary bovine mammary epithelial cells (BMECs) by a tissue explant culture method. When cell density reached 40~50% confluence, cells were isolated using 0.05% trypsin-0.02% EDTA. Then, the cells were diluted to aliquot into each well of 96-well plates to obtain the single clone primary BMECs. If primary BMECs’ density reached 40~50% confluence in each well of the 96-well plates, these cells further continued to amplify culture in 12-well plates; eventually, the pure primary BMECs were obtained. Next, the pure primary BMECs were seeded into six-well plates and reached 40% confluence. These cells were infected with a recombinant retrovirus expressing either the SV40T antigen or E6E7 containing 8 μg/mL polybrene, respectively. The six-well plate was centrifuged at 1200× *g* for 90 min at 32 °C to increase infection efficiency. After 12 h, the supernatants were removed and added in a fresh DMEM/F12 medium and began to screen the positive clone in 400 μg/mL G418 for 14 d. Finally, the immortalized BMECs were established. Immortalized BMECs were cultured in Dulbecco’s Modified Eagle Medium/Nutrient Mixture F12 (DMEM/F12; Gibco, Shanghai, China) supplemented with 10% fetal bovine serum (FBS; Gibco, Shanghai, China), 100 U/mL penicillin, and 100 µg/mL streptomycin (Sigma-Aldrich, Shanghai, China) at 37 °C with 5% CO_2_ atmosphere. All experiment protocols were complied with the guidelines of the Institutional Animal Care and Use Committee (IACUC) of Yang Zhou University.

### 2.2. Proliferation Assays

Cell proliferation assays were performed using the Cell Counting Kit-8 (CCK-8; Dojindo, Shanghai, China) according to the manufacturer’s protocol. Cells (6 × 10^2^) were seeded into 96-well tissue culture plates. After incubation, 10 µL CCK-8 reagent was added and cells were then incubated for 2 h. To determine the number of proliferated cells, absorbance was measured for each well at a wavelength of 450 nm, using an auto-microplate reader (Thermo Scientific, Shanghai, China).

### 2.3. Senescence-Associated β-Galactosidase Assay

Cell senescence assays were performed using the cell senescence β-galactosidase assay kit (Beyotime, Shanghai, China) according to the manufacturer’s protocol. Cells were seeded into six-well tissue culture plates. They were then washed with PBS and fixed in 3% formaldehyde for 15 min at room temperature. Cells were again washed with PBS and incubated in β-galactosidase staining solution overnight at 37 °C. Blue staining cells were observed with light microscopy, and images were taken at the same magnification. The quantification of SA-β-galactosidase-positive cells over the total cells was obtained by counting five random fields per well.

### 2.4. Illumina HiSeq mRNA Sequencing

Total RNA was extracted from BMECs using the TRIzol reagent (Invitrogen, Shanghai, China). The RNA (1 µg) of each sample with an RNA integrity number (RIN) value above 7 was used for a subsequent library preparation by Genewiz (Genewiz, Suzhou, China). Poly (A) mRNA isolation was performed using the NEB Next Poly (A) mRNA Magnetic Isolation Module (NEB, Beijing, China). The mRNA fragmentation and priming were performed using the NEB Next First Strand Synthesis Reaction Buffer and NEB Next Random Primers. First-strand cDNA was synthesized using the ProtoScript II Reverse Transcriptase (NEB, Beijing, China), and the second-strand cDNA was synthesized using a Second Strand Synthesis Enzyme Mix (NEB, Beijing, China). The purified double-stranded cDNA was then treated with an End Prep Enzyme Mix to repair both ends and add a dA-tailing in one reaction, followed by a T-aligation to add adaptors to both ends. Size selection of adaptor-ligated DNA was then performed using AxyPrep Mag PCR Clean-up (Axygen, Shanghai, China), and fragments of ~360 bp were recovered. Each sample was then amplified by 11 cycles of PCR using P5 and P7 primers. Both primers carried sequences that were able to anneal with flow cells to achieve bridge amplification, and the P7 primer carried a six-base index that facilitated multiplexing. PCR products were cleaned up using AxyPrep Mag PCR Clean-up (Axygen, Shanghai, China), validated using an Agilent 2100 Bioanalyzer (Agilent Technologies, Palo Alto, CA, USA), and quantified with a Qubit 2.0 Fluorometer (Invitrogen, Shanghai, China). A Kyoto Encyclopedia of Genes and Genomes (KEGG) pathway analysis was used to determine the significant pathways associated with the differentially expressed genes identified.

### 2.5. Western Blotting

Cells were lysed to extract total protein in a radioimmunoprecipitation assay buffer (50 mM Tris–HCl (pH 7.4), 150 mM NaCl, 1% NP-40, 0.1% SDS) containing 1× protease inhibitor buffer. Protein concentrations were determined using a BCA kit (Thermo Scientific, Shanghai, China). Equal amounts of protein lysates were fractionated by SDS-PAGE and transferred to nitrocellulose membranes (Pall, Shanghai, China). The membranes were blocked with 5% skimmed milk and then incubated with gentle shaking overnight at 4 °C, with the primary antibody plus 5% bovine serum albumin in Tris-buffered saline with Tween (TBS-T: 10 mM Tris–HCl, pH 7.5, 150 mM NaCl, 0.05% Tween 20). The following primary antibodies were obtained from Cell Signaling Technology (CST, Shanghai, China): rabbit anti-β-actin monoclonal antibody (diluted 1:1000; #4970), rabbit anti-GAPDH monoclonal antibody (1:1000; #2118), rabbit anti-cyclin D1 monoclonal antibody (1:1000; #2978), rabbit anti-CDK2 monoclonal antibody (1:1000; #2546), mouse anti-CDK6 monoclonal antibody (1:1000; #3136), rabbit anti-cyclin D3 monoclonal antibody (1:1000; #2936), rabbit anti-CDK4 monoclonal antibody (1:1000; #12790), and rabbit anti-p27^Kip1^ monoclonal antibody (1:1000; #3686). The following primary antibodies were obtained from Abcam company (Abcam, Shanghai, China): rabbit anti-E2F1 monoclonal antibody (1:1000; ab179445), rabbit anti-E2F2 monoclonal antibody (1:1000; ab138515), and mouse anti-p53 monoclonal antibody (1:1000; ab80644). The following primary antibodies were obtained from Santa Cruz Biotechnology (Santa Cruz, Shanghai, China): rabbit anti-E2F3 polyclonal antibody (1:200; sc-878), mouse anti-Cdc2 monoclonal antibody (1:200; sc-166885), and rabbit anti-p21^Cip1^ polyclonal antibody (1:200; sc-397). The horseradish peroxidase (HRP)-conjugated secondary antibodies used included goat anti-rabbit IgG (1:5000; #7074) and horse anti-mouse IgG (1:5000; #7076). All secondary antibodies were obtained from Cell Signaling Technology. The target bands were detected using the Super Signal West Femto Maximum Sensitivity Substrate or Pierce ECL Plus Western Blotting Substrate (Thermo Scientific, Shanghai, China).

### 2.6. Statistical Analysis

Statistical analysis was performed with one-way analysis of variance (ANOVA) followed by the least significant difference (LSD) test for post hoc multiple comparisons of treatment means, using SPSS 16.0 software (SPSS Inc.; Chicago, IL, USA). The *p*-values presented in the figures are represented as follows: * *p* < 0.05 and ** *p* < 0.01, and statistical significance was set at *p* < 0.05.

## 3. Results

### 3.1. SV40T- and E6E7-Induced Proliferation of BMECs

**Figure 1:**
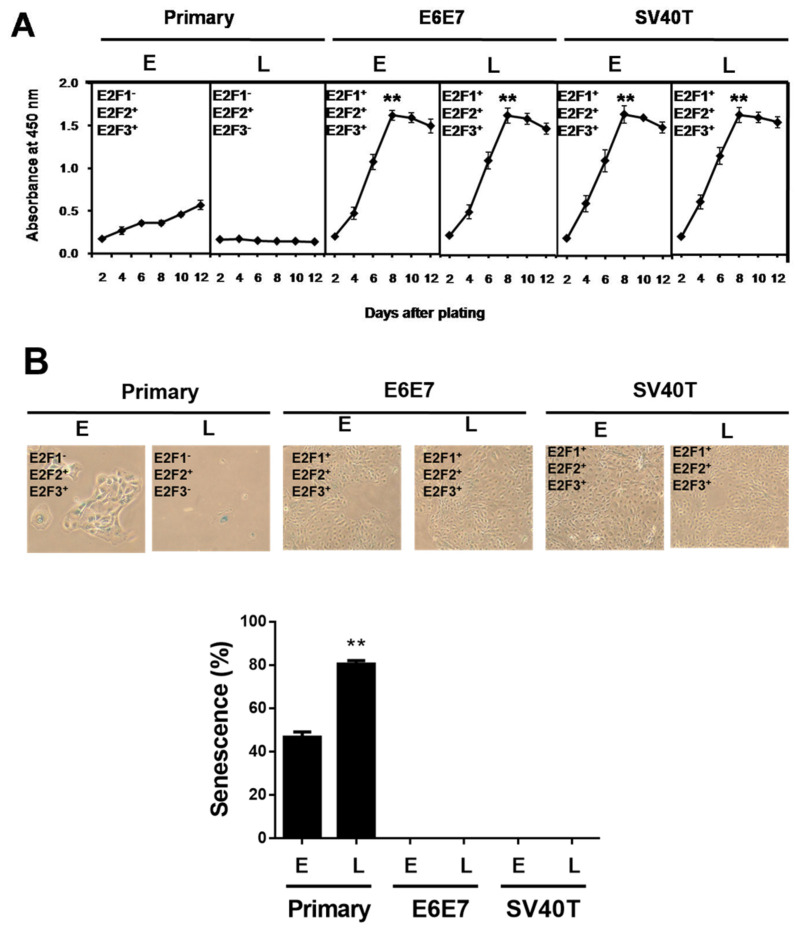
SV40T and E6E7 induce proliferation of BMECs containing E2F activators. (**A**) Growth curve comparison between primary and immortalized BMECs. Equal numbers of cells were cultured in a 96-well plate, and cells were periodically examined using CCK-8 in quadruplicate to obtain the growth curves (n = 4). ** *p* < 0.01 vs. primary BMECs. Primary BMECs or BMECs immortalized by E6E7 and SV40T were collected at early (E) and late (L) passages of culture. (**B**) Senescence-associated β-galactosidase staining to determine the status of senescence in primary and immortalized BMECs. Blue positive staining and large, flat morphology were observed in primary BMECs. Blue β-galactosidase staining was not observed in E6E7 and SV40T immortalized BMECs. The graph represents the percentage of senescent cells. ** *p* < 0.01 vs. primary early and immortalized BMECs. Primary BMECs or BMECs immortalized by E6E7 and SV40T were collected at early (E) and late (L) passages. The early and late stages of primary BMECs represent passages 10 and 30 of primary BMECs’ cultures, respectively. The early and late stages of immortalized BMECs represent passages 60 and 100 of immortalized BMECs’ cultures, respectively. Data are based on triplicate experiments.

Previously, we established two types of immortalized BMECs’ lines using SV40T or E6E7 [17]. The immortalized BMECs at a late stage (after 100 passages of culture) still maintained the same potential of proliferation as the cells at an early stage, while the primary BMECs lost the ability to proliferate at a late stage (Figure 1A). To determine whether the ability of proliferation is associated with senescence, β-galactosidase staining assays were carried out in the primary and immortalized BMECs. As expected, the percentage of senescent cells at the late stage of primary culture showed as markedly greater than at a primary early stage (*p* < 0.01), and the immortalized ones did not (Figure 1B).

### 3.2. Identification of Signaling Pathways Responsible for BMECs’ Immortalization by Transcriptome Analysis

**Figure 2:**
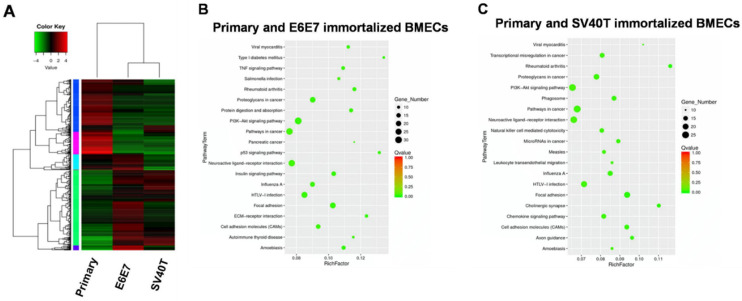
Transcriptome analysis for signaling pathways in primary and immortalized BMECs. (**A**) Hierarchical heat map showing the expression values for transcript mRNA in primary and immortalized bovine mammary epithelial cells (BMECs). Red indicates higher expression and green indicates lower expression. (**B**,**C**) KEGG pathway enrichment scatter diagram for differentially expressed genes. The range in the circle represents the number of differentially expressed genes in each pathway.

To address the mechanism underlying the proliferation and senescence of the BMECs induced by SV40T and E6E7, we performed transcriptome analysis of the primary and immortalized BMECs. Transcriptome analysis of primary and immortalized BMECs revealed a marked difference in the gene expression profile between the two types of cells, but the two immortalized BMECs shared a similar pattern of expression (Figure 2A). Pathway analysis showed that the genes differentially expressed in primary and E6E7-immortalized BMECs were significantly enriched in the ‘pathways in cancer’, ‘PI3K-Akt signaling pathway’, and ‘neuroactive ligand–receptor interaction’ (Figure 2B). In line with the gene expression profiling analysis, the three pathways for primary and E6E7-immortalized BMECs were also revealed by pathway analysis on the primary and SV40T-immortalized BMECs (Figure 2C). As the ’pathways in cancer’ and ‘PI3K-Akt signaling pathway’ are involved in cell proliferation, the genes in these pathways may be responsible for BMECs’ proliferation.

### 3.3. Involvement of E2F Signaling Pathway in the Proliferation of the Immortalized BMECs

**Figure 3:**
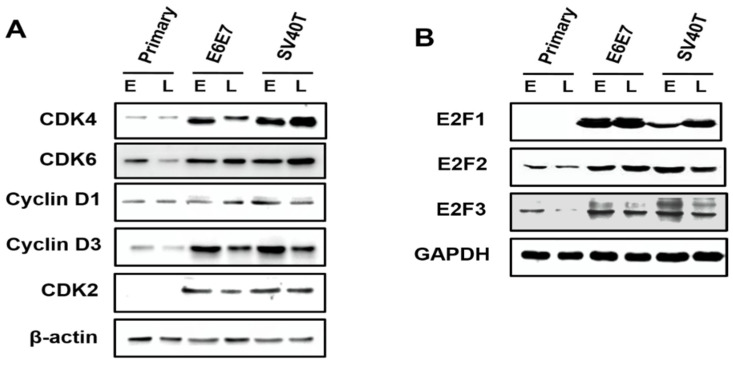
Involvement of E2F signaling pathway in the proliferation of the immortalized BMECs. (**A**) Western blotting analysis of CDK4, CDK6, cyclin D1, cyclin D3, and CDK2 in primary and immortalized BMECs. (**B**) Western blotting analysis of E2F1, E2F2, and E2F3 in primary and immortalized BMECs. Primary BMECs or BMECs immortalized by E6E7 and SV40T were collected at early (E) and late (L) passages of culture. Data are based on triplicate experiments.

Analysis on the expression of the cyclin-dependent kinases (CDKs) in primary and immortalized BMECs indicated that, compared to primary cells, the CDK4 and CDK6 were enhanced in SV40T- and E6E7-immortalized BMECs, compared to primary BMECs (Figure 3A). Similarly, it was found that the expression of CDK2 was elevated in immortalized BMECs, while the CDK2 protein was not expressed in the primary BMECs (Figure 3A). As cyclin can form a complex with and functions as a regulatory subunit of CDKs, a high expression of cyclin can promote cell proliferation. Indeed, Western blotting analysis showed that the expression of cyclin D3 was significantly higher in the immortalized BMECs than the primary ones. In contrast, there was no obvious difference in the expression of cyclin D1 across the cells (Figure 3A). Together, these findings suggested that CDK4, CDK6, CDK2, and cyclin D3 were the molecules responsible for the proliferation of BMECs induced by SV40T and E6E7.

The E2F2 and E2F3 can be expressed at an early stage of primary BMECs, but E2F1 was not found across the primary BMECs (Figure 3B). Obviously, the E2F2 is also expressed at a late stage of primary BMECs; however, the E2F3 protein is lost at a late stage of primary BMECs’ culture (Figure 3B). Compared with the late stage of primary BMECs, the BMECs immortalized by SV40T and E6E7 restored the protein level of E2F3, indicating that E2F3 plays a vital role in the proliferation of BMECs (Figure 3B).

### 3.4. Involvement of p53 Signaling Pathways in the Proliferation of the Immortalized BMECs

**Figure 4:**
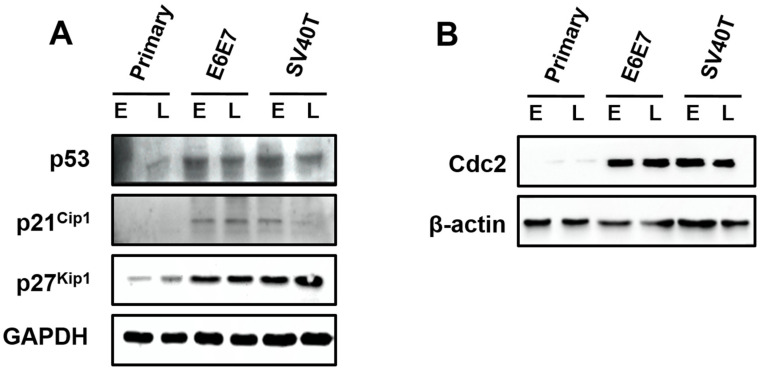
Involvement of p53 signaling pathways in the proliferation of the immortalized BMECs. (**A**) Western blotting analysis of p53, p21Cip1, and p27Kip1 in primary and immortalized BMECs. (**B**) Cdc2 kinase assays of primary and immortalized BMECs. Primary BMECs or BMECs transduced by E6E7 or SV40T were collected at early (E) and late (L) passages of culture. Data are based on triplicate experiments.

Surprisingly, the expression of p53, p21^Cip1,^ and p27^Kip1^ were induced by SV40T/E6E7 in the immortalized BMECs, relatively to primary ones (Figure 4A). The Cdc2 was almost expressed in the primary BMECs, but the BMECs immortalized by SV40T or E6E7 led to an elevation of Cdc2 (Figure 4B). As inhibition of p53, p21^Cip1^, and p27^Kip1^ are generally associated with cell proliferation, it was unlikely that these proteins were important to the proliferation of the immortalized BMECs. However, Cdc2 in the p53 signaling pathway seemed to play a key role in the proliferation of the immortalized BMECs, suggesting the expression of Cdc2 was subjected to regulation by other molecules than p53. In addition, the protein expression of Cdc2 appeared to be associated with the senescence of the primary BMECs (Figure 4B).

## 4. Discussion

Previous studies have demonstrated that disruption of the E2F and p53 tumor suppressor pathways occur in human tumorigenesis [6]. One of the critical steps in this process is immortalization of the cells by tumor suppressor factors and oncogenes that facilitate the bypass of G1–S–M phases [18]. Replicative senescence is commonly accompanied by the downregulation of CDKs [19]. In addition, E6E7 and SV40T have the capacity to cause the release of E2F activators that drive cell cycle progression by increasing the levels of CDKs required for entering G1 into the S-phase [4]. As the E2F signaling pathway plays a critical role in cell proliferation [4,20], and the E2F pathway was a part of the ‘pathways in cancer’ identified in this study, we speculated that the E2F pathway contributed to BMECs’ proliferation. To confirm this speculation, the protein expression of genes related to the E2F pathway (i.e., CDK2/4/6, Cyclin D1/3, E2F1/2/3, and Cdc2) was determined by Western blotting analysis in the immortalized and primary BMECs at early and late stages of culture. The CDK4 and CDK6 are important molecules upstream of E2F in the signaling pathway [21,22]. In this study, we demonstrated that a specific E2F3 activator is required to induce cellular proliferation in BMECs immortalized by E6E7 and SV40T. The complex of cyclin D-CDK4/6 is responsible for the cell proliferation and cell cycle progression at the G1 phase [23,24]. In addition, cyclin E in combination with CDK2 is able to bypass the S phase checkpoint [19,25]. The activity of CDKs can regulate the E2F transcription factor; this can then promote the expression of various genes related to the entry of cells into the S phase [26]. Based on our present results, CDK4, CDK6, cyclinD1, and cyclin D3 exhibit the low protein levels at the early and late stages of primary BMECs. Particularly, CDK2 cannot be expressed at a total stage of primary BMECs’ culture. Primary early BMECs containing the low expression of E2F2 and E2F3 still proliferated, although the protein expression of E2F1 was missing at the early stage of primary BMECs. The result indicated that the proliferation of BMECs is not related to E2F1 at the stage of primary culture. More specifically, primary late BMECs lacking the E2F3 completely abrogated proliferation, showing a large, flat morphology and β-galactosidase activity. In addition, the BMECs immortalized by E6E7 and SV40T restored the high E2F3 protein levels; this, in turn, drives the proliferation of BMECs and cell cycle progression. These results demonstrated that the E2F3 plays a central role in the proliferation of BMECs. In contrast to a recent study, SV40T antigen-induced proliferation is not required for the depletion of the E2F3 activator in MEFs [8]. Although transformation of SV40T antigen-induced MEFs occurs in the absence of E2F1, E2F2, and E2F3 activators, these cells still show robust proliferation [7]. Furthermore, epithelial stem cells and developing progenitors normally enter the cell cycle triply deficient in E2F1, E2F2, and E2F3 [12]. These contrasting results indicate that both E2F3-dependent and -independent pathways result in cell cycle entry and proliferation, possibly depending on the cell type and background conditions.

It is known that CDKs/cyclin D complex is subjected to the regulation of p21^Cip1^ and p27^Kip1^, which function as inhibitors of cell proliferation or regulators of cell cycle in the PI3K/AKT and p53 signaling pathways [27,28,29,30]. Importantly, both pathways were part of the ‘pathways in cancer’. We thus determined the expression of some genes (i.e., p53, p21^Cip1,^ p27^Kip1^, and Cdc2) in the pathways by Western blotting analyses. To validate relevant downstream genes that might be responsible for the control of cellular growth arrest due to the loss of the E2F3 activator, we examined the E2F3 target gene and status of the p53 pathway in addition to various inhibitors of the cell cycle, including p21^Cip1^ and p27^Kip1^. The levels of p21^Cip1^ were undetectable at the stages of primary culture. Interestingly, p21^Cip1^ and p27^Kip1^ were expressed in E6E7- and SV40T-immortalized BMECs. These results suggest that p21^Cip1^ and p27^Kip1^ are not likely essential for p53 pathway target genes in this context. More importantly, the Cdc2 involved in the E2F3 target gene was not found at the total stages of primary culture and severely impaired the BMECs’ proliferation. However, the E6E7 viral protein and SV40T antigen could enhance Cdc2 overexpression and cell proliferation. Additionally, the ectopic expression of Cdc2 could bypass the G2/M phase checkpoint to escape programmed cell death. Altogether, these findings support the idea that SV40T/E6E7-induced proliferation is not involved in the p53 signal pathway in BMECs.

## 5. Conclusions

In conclusions, at an early stage, primary BMECs lacking the E2F1 expression and containing E2F2 and E2F3 slight protein levels have the capacity to proliferate, indicating that E2F1 expression is not involved in the BMECs’ proliferation. Although the E2F2 can be expressed at a late stage of primary BMECs, primary BMECs deficient for E2F3 completely abolish any proliferative ability at a late stage. In addition, the BMECs immortalized by SV40T and E6E7 restored the protein level of E2F3. Overall, our study demonstrates that E2F3 plays a central role in the proliferation of BMECs.

## Data Availability

Not applicable.
